# Convergence of Electronic Structure Properties in Ionic Oxides Within a Fragment Approach

**DOI:** 10.3389/fchem.2022.951144

**Published:** 2022-07-15

**Authors:** Ernst D. Larsson, Valera Veryazov

**Affiliations:** Division of Theoretical Chemistry, Chemical Centre, Lund University, Lund, Sweden

**Keywords:** ionic solids, ab initio model potential, embedded clusters, fragment approach, electronic structure, valency

## Abstract

Embedded-cluster models of crystalline solids are important to allow accurate wave function methods to be applicable to solids. The ab-initio model potential method, in which the crystal is divided into three different fragments, one quantum fragment, one ab-initio model potential fragment and one point-charge fragment, has historically been shown to be a viable tool for describing the electronic structure in ionic solids. The optimal size of these regions is, of course, individual for each crystal. In this study we analyzed the convergence of the electronic structure properties with respect to an increase of the size of the quantum part and the layer of potentials. *MgO* crystal and *Ni*: *MgO* were used for this purpose as examples of an ideal crystal and a crystal with a point defect. We demonstrated that with an increase of the cluster size, the electron density in the inner part of the cluster becomes very similar to the electron density in the periodic model. Clusters, embedded into a layer of model potential and electrostatic field, are a good alternative to periodic description.

## 1 Introduction

Metal oxides are important materials with many industrial applications in the areas of catalysis, glass manufacture, extractive metallurgy and many others (Jolivet et al., 2000; Di Cosimo et al., 2014; Arunadevi and Kirubavathy, 2022). Many metal oxides are characterized by highly ionic chemical bonds, which plays an important role in the properties of the materials and chemical reactions.

Alkali and alkaline earth metal oxides are good insulators with a very large optical gap, which makes them an ideal candidates for treatment by means of band theory in combination with density functional theory (DFT) (Schönberger and Aryasetiawan, 1995; Canney et al., 1999; Lany, 2014; Heo et al., 2015; Niedermeier et al., 2016). The interest to small alkaline earth metal oxides clusters is stimulated by a potential use of these materials for hydrogen storage (Srivastava et al., 2018; Mojica-Sánchez et al., 2019) and in biomedical applications (Awasthi et al., 2021). In the presence of dopants, for example, transition or rare metals in metal oxides (these compounds are most interesting from a scientific and industrial point of view), the electronic structure is much more complicated. First of all, in order to model a sparse distribution of the dopants in the host material, the unit cell has to be substantially increased to a so-called supercell. The accuracy of DFT might be insufficient to reproduce the electronic structure of highly correlated d- and f-elements. For these materials, the embedded cluster approach can be considered as an alternative to band structure calculations: 1) there is more flexibility in the selection of the size and the shape of a cluster, 2) the electronic structure calculations are not limited by DFT, but instead the full arsenal of accurate molecular methods from coupled cluster to multiconfigurational theory can be applied. These accurate methods provide a consistent way to include electron correlation effects, but only methods based on a single reference wavefunction were recently generalized for the periodic boundary conditions (Ayala et al., 2001; Hirata et al., 2004; McClain et al., 2017).

At the same time, DFT makes it possible to perform a detailed comparison between periodic models and cluster models of increasing size. Ideally, the inner part of a cluster should have the same electron density as in an infinite crystal. An increase of the cluster size should make the difference between the reference electron density (as in periodic model) and the electron density in the cluster smaller and smaller. In the current study we considered two systems: magnesium oxide (*MgO*) and magnesium oxide with sparse nickel substitution of magnesium atoms (*Ni*: *MgO*). Pure *MgO* is a wide gap insulator and thus one can expect very small dependence of the results on the amount k-points (or, similarly on the size of the supercell for a calculation with only the *Γ* point). For the cluster models, assuming non stoichiometric clusters made by the neighboring layers of atoms, there are two possibilities - clusters that are either centered at a magnesium atom or at an oxygen atom. Modeling sparse defects is different from modeling ideal crystals. Substitution of *Mg* to *Ni* in the cluster model (centered at magnesium atom) is a rather trivial change, but for periodic models it is necessary to increase the size of the supercell, in order to remove or reduce the interaction between dopant atoms.

The drawback of the cluster approach in electronic structure calculations is the cluster border. Using bare clusters, such as ${\left[MgO_{6}\right]}^{10-}$ , without compensating the large formal charge, is insufficient for ionic crystals. Such clusters frequently display poor convergence patterns and might result in unphysical charge distributions. Placing a cluster into an electrostatic field in order to reproduce the Madelung potential from the infinite crystal, is not sufficient because of the presence of partially covalent bonds even in highly ionic crystals and potential “electron-leakage” effects Ref. Shi et al. (2022). Thus a typical solution for an embedding will include both terminating atoms or specially prepared potentials and an electrostatic field. We recently suggested a new protocol for computing model potentials, which can be used at the border of ionic clusters Larsson et al. (2022). In the current work, we will investigate the convergence of properties, related to the electronic structure, with increasing cluster sizes. For reference we will use band structure calculations, made with the same basis set and same DFT functional as in the cluster models. Although we focus on the proof that the electron density in the central part of embedded cluster is close to the electron density in periodic calculations at DFT level, our final goal is to apply accurate ab initio methods can be used to study the ground and excited states.

## 2 Materials and Methods

Selection of clusters for ionic crystals is not an easy task. Stoichiometric clusters have complicated border structures and are generally not suitable for systems with a point defect. Clusters constructed by adding spheres of neighboring atoms starve from having an uncompensated electronic charge in the quantum region. Taking a cluster such as ${\left[MgO_{6}\right]}^{10-}$ as an example again, adding only point charges will only partially compensate the large formal charge, since the electron density in the quantum region can unphysically spread into the point charge region. In order to compensate for the effect of broken bonds at the border of the cluster, a separate layer can be introduced to the model. For covalent systems, a typical solution will include the use of terminating hydrogen atoms, while for ionic systems the termination can be achieved by placing specially prepared model potentials. These potentials can mimic the absent atoms, so the electron density inside the quantum region will be unperturbed by the cluster border. The presence of these potentials in the calculation only slightly increases the computational costs, since there are not necessarily basis set functions associated with these potentials.

The most reliable and straightforward method for construction of these potentials is AIMP (ab initio model potential) (Seijo and Barandiarán, 1999; Seijo and Barandiarán, 2013). In the Hartree-Fock equation, the Fock operator, $\hat{f}$ , is replaced by ${\hat{f}}^{AIMP}=\hat{f}-2\underset{k}{\sum }\epsilon_{k}|\psi_{k}\rangle \langle \psi_{k}|$ . Originally, the index *k* was used only to describe the core-states, making AIMPs a type of effective core potentials. Later, it was realised that by design, the AIMP methodology could be use to also freeze valence orbitals, leading to their use as embedding potentials Ref. Barandiarán and Seijo, (1988), Seijo and Barandiarán, (1999). Thus, in an embedded cluster calculation, *k* runs over all occupied orbitals of the ion described as an AIMP. The one electron energies of the states in the original formulation of AIMPs are defined by Hartree-Fock calculations. In Ref. Larsson et al. (2022) we demonstrated that it is also possible to use one electron energies obtained by DFT calculations, in particular with the PBE functional. The practical limitation of the method is related to the fact that these potentials depend on the crystal structure and should be recomputed for each new crystal structure. We recently suggested a procedure for such a purpose: a code named SCEPIC Larsson et al. (2022) allows one to construct the model potentials based on either HF or DFT. Using this code we constructed model potentials for several ionic crystals and demonstrated that the best quality for the embedding, at least for pure crystals, can be obtained with modified AIMPs, where the construction of the potentials uses PBE-based one electron energies. In the current study we will use the same model potentials, since the structure of the crystal is the same as it was used in Ref. Larsson et al. (2022). In addition to the layer of AIMPs, a set of point charges should be implemented to provide electroneutrality of the whole system. For the point charges, modeling an electrostatic field from the infinite crystal, we used the same strategy as in Sushko and Abarenkov, (2010): a finite set of charges were generated using an algorithm from Ref. Abarenkov, (2007).

The *MgO* crystal has a rock-salt structure with 6 oxygen atoms surrounding a magnesium atom. We can build a set of clusters, centered on an atom, by adding the layers from the next neighbours. The smallest cluster has a composition *MgO*_6_. This cluster formally has a large electronic charge, however this charge will be compensated by terminating model potentials at the border and by a set of point charges, simulating the Madelung potential of a *MgO* crystal. Adding the next layer of neighboring atoms, we will get a cluster with composition *MgO*_6_ *Mg*_18_, *MgO*_6_ *Mg*_18_ *O*_38_ and finally *MgO*_6_ *Mg*_18_ *O*_38_ *Mg*_66_. One should not expect monotonic changes in local properties for these incrementally built clusters, since the electronic charge, associated with the quantum part, as well as the atoms on the border of the cluster are alternating between *Mg* and *O* depending on cluster size. The nomenclature of these clusters will be on the form ${\left[MgO_{6}Mg_{18}\right]}^{26+}$ etc. to emphasize that the additional ions are, strictly speaking, not equivalent to the inner ions. Occasionally, a more extensive nomenclature on the form ${\left[Mg^{\left(i\right)}O_{6}^{\left(i\right)}Mg_{18}^{\left(o\right)}\right]}^{26+}$ , when necessary to distinguish between inner (*X* ^(*i*)^), outer (*X* ^(*o*)^) and “extra”(*X* ^(†)^) ions. Table 1 introduces a short notation for the clusters, and specifies the details of partitioning: the quantum size, the amount of AIMPs and point charges. The increase of size of these clusters exposes an important problem in quantum chemistry. Accurate computational methods scale poorly with respect to the increase of the basis set size. While DFT-like methods scale as *N* ^3^ − *N* ^4^, where *N* is the size of the basis set, multiconfigurational methods scale as *N* ^7^. Table 1 also contains the number of basis functions for the PC-1 basis set (used in the current study in order to reach clusters with as many as possible layers) and for a triple-*ζ* quality basis set (ANO-RCC-VTZP), which should be used for accurate calculations.

**TABLE 1:** Notations for the clusters, used in the study, indicating the quantum part, AIMPs and point charges. The basis set size is presented for PC-1 (used in the current study) and for a triple-*ζ* quality basis set.

Notation	Quantum Part	Formal	AIMPs	*#* Of Point	Basis Set Size	
		charge		charges	PC-1	VTZP
**Mg-I**	$\left[Mg^{\left(i\right)}O_{6}^{\left(i\right)}\right]$	−10	$\left[Mg_{54}^{AIMP}O_{56}^{AIMP}\right]$	51986	94	214
**Mg-II**	$\left[Mg^{\left(i\right)}O_{6}^{\left(i\right)}Mg_{18}^{\left(o\right)}\right]$	+26	$\left[Mg_{122}^{AIMP}O_{110}^{AIMP}\right]$	51846	274	826
**Mg-III**	$\left[Mg^{\left(i\right)}O_{6}^{\left(i\right)}Mg_{18}^{\left(o\right)}O_{38}^{\left(†\right)}\right]$	−50	$\left[Mg_{230}^{AIMP}O_{192}^{AIMP}\right]$	51618	806	1966
**Mg-IV**	$\left[Mg^{\left(i\right)}O_{6}^{\left(i\right)}Mg_{18}^{\left(o\right)}O_{38}^{\left(†\right)}Mg_{66}^{†}\right]$	+82	$\left[Mg_{368}^{AIMP}O_{422}^{AIMP}\right]$	51184	1466	4210
**O-I**	$\left[O^{\left(i\right)}Mg_{6}^{\left(i\right)}\right]$	+10	$\left[O_{54}^{AIMP}Mg_{56}^{AIMP}\right]$	51986	74	234
**O-II**	$\left[O^{\left(i\right)}Mg_{6}^{\left(i\right)}O_{18}^{\left(o\right)}\right]$	−26	$\left[O_{122}^{AIMP}Mg_{110}^{AIMP}\right]$	51846	326	774
**O-III**	$\left[O^{\left(i\right)}Mg_{6}^{\left(i\right)}O_{18}^{\left(o\right)}Mg_{38}^{\left(†\right)}\right]$	+50	$\left[O_{230}^{AIMP}Mg_{192}^{AIMP}\right]$	51618	706	2066
**Ni-I**	$\left[Ni^{\left(i\right)}O_{6}^{\left(i\right)}\right]$	−10	$\left[Mg_{54}^{AIMP}O_{56}^{AIMP}\right]$	51986	115	239
**Ni-II**	$\left[Ni^{\left(i\right)}O_{6}^{\left(i\right)}Mg_{18}^{\left(o\right)}\right]$	+26	$\left[Mg_{122}^{AIMP}O_{110}^{AIMP}\right]$	51846	295	851
**Ni-III**	$\left[Ni^{\left(i\right)}O_{6}^{\left(i\right)}Mg_{18}^{\left(o\right)}O_{38}^{\left(†\right)}\right]$	−50	$\left[Mg_{230}^{AIMP}O_{192}^{AIMP}\right]$	51618	827	1991
**Ni-IV**	$\left[Ni^{\left(i\right)}O_{6}^{\left(i\right)}Mg_{18}^{\left(o\right)}O_{38}^{\left(†\right)}Mg_{66}^{†}\right]$	+82	$\left[Mg_{368}^{AIMP}O_{422}^{AIMP}\right]$	51184	1487	4235

Doping ionic metal oxides with transition metals or rare earth metals creates materials with interesting physicochemical properties. Many of these materials are active in chemical reactions, including catalytic. In practically interesting cases, the concentration of impurities is rather low. *Ni*: *MgO* is a compound where a *Ni* atom substitutes a *Mg* atom. Since the ionic radii of *Mg* ^2+^ and *Ni* ^2+^ are similar, we can construct corresponding clusters similar to those mentioned above with the center in magnesium atom. The smallest cluster, *NiO*_6_, has no *Mg* atoms at all, but the AIMP layer, as earlier, contains model potentials for magnesium and oxygen. The larger clusters will contain layer(s) of magnesium. In periodic model it is important to use relatively large supercells to provide a spatial separation between the defects.

**FIGURE 1:**
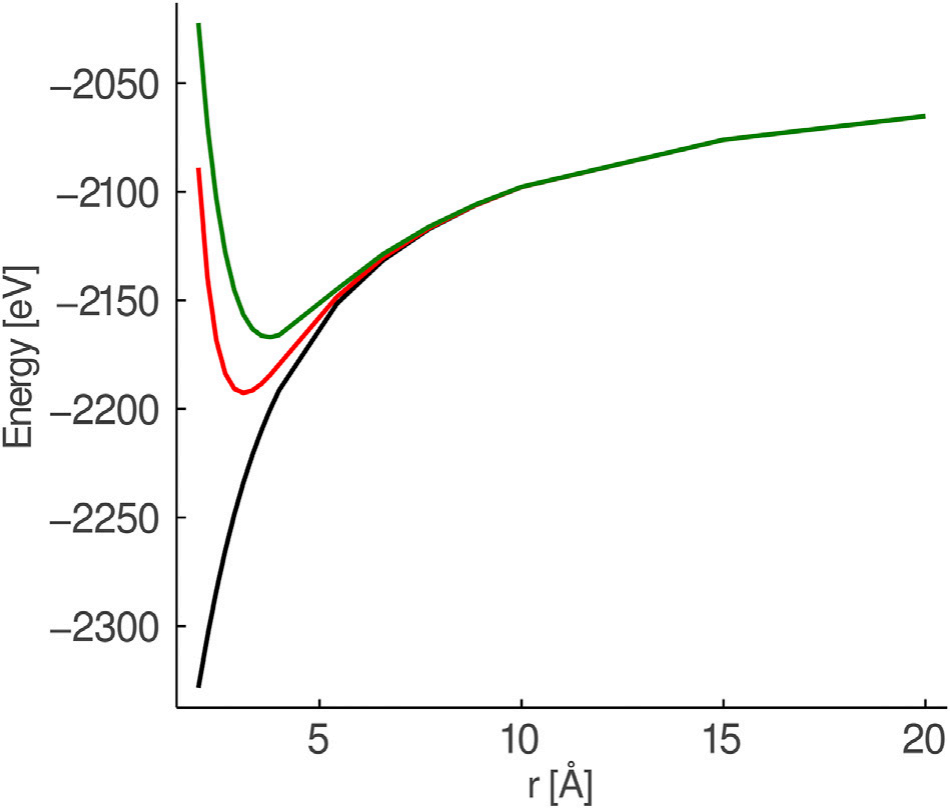
Comparison of the potential from a set of six point charges (black), six *Mg* ^2+^-AIMPs (red) and six *Ca* ^2+^-AIMPs (green) surrounding a *O* ^2−^-ion. The surrounding charges/AIMPs are placed in an octahedral field and stretched symmetrically.

Each cluster was surrounded by AIMPs up to a cutoff radius of the longest distance between two real atoms in the QM region plus the length of one unit-cell (4.26 Å) surrounding the central cluster, making the cluster plus AIMP-region spherical in shape. For instance, in ${\left[MgO_{6}\right]}^{10-}$ , the longest distance is 2.13 Å (i.e., the *Mg*–*O* bond length in *MgO*), making the radius of the sphere 6.39 Å. This was made primarily for convenience; Ref Larsson et al. (2022) showed that the cluster region should be surrounded by at least one unit-cell layer of AIMPs for local properties to converge. This is further supported by Figure 1, which shows that at a range of beyond c:a 6 Å, AIMPs start to behave like a classical charge with a finite volume. Therefore, having more AIMPs than is necessary does not influence the results of the calculation, only the evaluation of the one-electron Hamiltonian which becomes somewhat slower with increasing the number of AIMPs Ref Larsson et al. (2022). Since they behave primarily as charges beyond a certain range, the total charge of the AIMP region bears no influence on the calculation, and the only important charge to consider is that of the QM region itself. The total structure including the point-charge region was constructed using a radius of 50 Å; the total structure is, however, not a sphere, since with the GPEE method, GPEE-modified *unit-cells* up to a range of 50 Å were included, rather than *ions* up to that radius. Multipole moments up to third order were canceled by the GPEE method.

**FIGURE 2:**
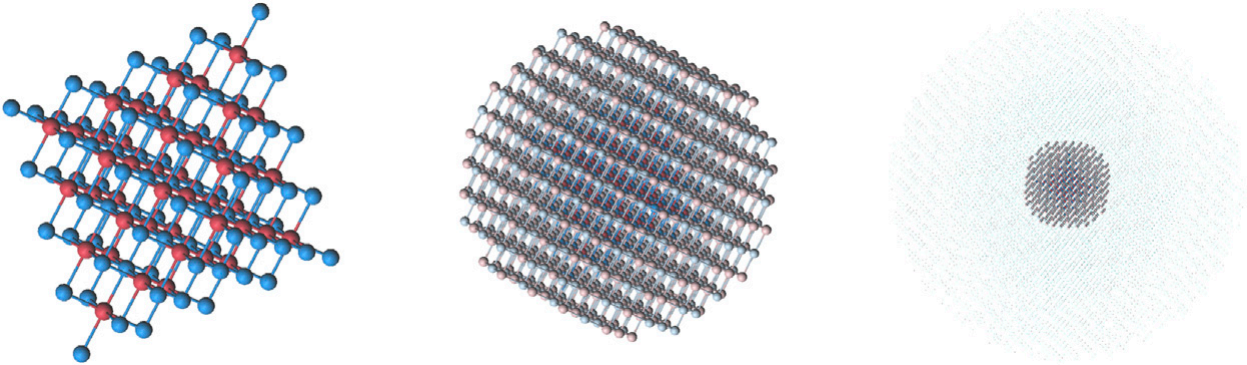
The structure of Mg-IV cluster. From left to right: the quantum part, AIMPs layer (model potentials are shown as pale spheres), point charges surrounding the embedded cluster.

For the smallest cluster, ${\left[MgO_{6}\right]}^{10-}$ , the procedure described in the previous paragraph leads to an AIMP region of ${\left[Mg_{54}^{AIMP}O_{56}^{AIMP}\right]}^{4-}$ and point-charge region with a total charge of +14 e in order to provide an electroneutrality of the whole system. The AIMP region increased to ${\left[Mg_{122}^{AIMP}O_{110}^{AIMP}\right]}^{24+}$ for the ${\left[MgO_{6}Mg_{18}\right]}^{26+}$ -cluster, with the point-charge region having a total charge of -50 e. Next, ${\left[MgO_{6}Mg_{18}O_{33}\right]}^{50-}$ , an AIMP region of ${\left[Mg_{230}^{AIMP}O_{192}^{AIMP}\right]}^{76+}$ and point-charge region of total charge -26 e was used. And finally the largest cluster is ${\left[MgO_{6}Mg_{18}O_{33}Mg_{66}\right]}^{82+}$ with an AIMP region ${\left[Mg_{368}^{AIMP}O_{422}^{AIMP}\right]}^{108-}$ and point-charge region +26 e. For these clusters we used nomenclature **Mg-I**, **Mg-II**, **Mg-III** and **Mg-IV**, where the Roman number specifies the number of *Mg* spheres, as shown in Table 1. At Figure 2 the structure of **Mg-IV** cluster is presented in a visual form: the quantum part, the layer of AIMPs and finally a cloud of point charges. For **Mg-IV** cluster calculations the disk space for temporary storage was about 210Gb, making further increases of the cluster size unfeasible. The *O*-centered clusters are identical in construction to the *Mg*-centered, and can be derived by simply inverting the positions of *Mg* and *O* and changing the signs of all charges. The nomenclature of these clusters is **O-I**, **O-II** and **O-III**. The largest cluster with a center at an oxygen atom (**O-IV**) is not included in the results, because in this case we did not manage to obtain a stable solution for the wavefunction calculation. *Ni*-centered clusters were obtained by simply replacing the central *Mg* with *Ni*, without any changes in geometry. The nomenclature for these clusters is from **Ni-I** to **Ni-IV**.

All electronic structure calculations in this work utilized the Perdew–Burke–Ernzerhof (PBE) DFT functional with the pc-1 basis set Jensen, (2002). Cluster calculations were performed with OpenMolcas, with the embedding method described in Ref. Larsson et al. (2022). Periodic calculations were made with CP2K, using all-electron basis sets within the Gaussian and augmented plane wave (GAPW) framework, with a plane wave cutoff of 400 eV. The periodic calculations were based only on the *Γ*-point, using both a 2 × 2 × 2 supercell and a 3 × 3 × 3 supercell.

### 2.1 Electron Density Comparison

The electron density is a direct and a straightforward way to compare different cluster and periodic model calculations. Ideally, the electron density in a large enough cluster should be identical or close enough to the electron density obtained from a periodic calculation. Unphysical effects arising from the cluster–embedding border can be very strong and influence the central part of the quantum region. Simply increasing the cluster size might, however, not necessarily improve upon the results, as the number of broken bonds at the border of the cluster increases with cluster size. In principle, a large enough bare cluster would be the most realistic model of an ionic crystal. Unfortunately, the size required to reproduce the Madelung field from the extended crystal will in general prohibit such a route. One way to circumvent these problems is to use specially constructed potentials, designed to behave as similar as possible to the real atoms, at the border between the quantum region and the electrostatic embedding.

There are two options for comparing the electron density. First option includes the comparison of so-called local properties of the electronic structure: charges (*Q*), bond orders (*W*), covalency (*C*) and total valency (*V*). All these properties are computed from the population matrix (**DS**), where **D** is the density matrix and **S** is the overlap matrix. For molecular and cluster calculations the size of the matrix is determined by basis set size, but for periodic calculations it is determined by the basis set size in the supercell used in the calculation Veryazov et al. (1999). Although these properties depend on the atomic basis set, with a fixed basis set they can be used as an indicator of similarities between two electron densities. Atomic charges defined as a number of electrons, involved in ionic bonding and it is defined as a difference between nuclear charge *Z*_*A*_ and the electron charge localized at the atomic region $\left(Q_{A}=Z_{A}-\underset{a\in A}{\sum }{\left(DS\right)}_{ii}\right)$ , where *a* is a molecular orbital from atom *A*. The bond order between atoms A and B $\left(W_{AB}=\underset{a\in A}{\sum }\underset{b\in B}{\sum }|{\left(DS\right)}_{ij}{|}^{2}\right)$ corresponds to the number of electrons involved in the bond between the atoms Wiberg, (1968). Covalency $\left(C_{A}=\underset{B\neq A}{\sum }W_{AB}\right)$ counts the number of electrons involved in covalent bonds Armstrong et al. (1973), and finally, the total valency ( $V_{A}=\frac{1}{2}\left(C_{A}+\sqrt{C_{A}^{2}+4_{*}|Q_{A}{|}^{2}}\right)$ is a property, which counts all electrons from a certain atom, that are involved in the creation of chemical bonds in the system Evarestov and Veryazov, (1991).

To compute local properties from the wavefunctions produced by OpenMolcas and CP2K we used homemade scripts. For ionic crystals atomic charges are similar to formal charges, but never perfectly match them. The contributions to the covalent bonds are small and local: all bond indices between non-neighboring atoms are close to zero. It is important to compare the combination of all local properties to confirm the conclusion about the match between different electron densities.

Another alternative is to use spatial electron density *ρ*(*r*). Both OpenMolcas and CP2K can produce spatial electron density computed on a Cartesian grid. Since the electron density is a function of coordinates, the comparison can be performed in some arbitrary volume. For that purpose we used the union of seven spheres centered on the atomic positions of the *MgO*_6_ cluster, with radii of 1.6Å (3/4 of *MgO* bond length). This volume represents the density in the central part of the studied clusters, however for the smallest clusters (**Mg-I**, **O-I**) the border of this volume is too close to the cluster border. Different computational codes can use different approximations in computing electronic density in the regions close to atoms, for instance, the GAPW framework in CP2K, used in all-electron calculations, separate the electron density into a core (hard) density and valency (soft) density Ref Hutter et al. (1999). Only the valence region is dealt with in a similar fashion to standard molecular quantum chemistry codes, such as OpenMolcas. Thus, we removed spherical regions with the radius 0.8Å around each atom, which corresponds to the default value for the core density in CP2K.

Using Cartesian grid in the volume described above, we introduce the root mean square deviation (RMSD) of the density: $\rho^{RMSD}=\sqrt{\frac{\sum_{i}^{n}{\left(\rho_{i}^{\text{cluster}}-\rho_{i}^{\text{periodic}}\right)}^{2}}{n}}$ , where the index *i* runs over all points on a grid, which is about 200000 points.

## 3 Results and Discussion

### 3.1 Local Property Descriptors in MgO

In this section we present the results for clusters centered on magnesium (**Mg-I**, **Mg-II**, **Mg-III** and **Mg-IV**) and clusters centered on oxygen (**O-I**, **O-II**, **O-III**). Calculation of the electronic structure of **O-IV** cluster diverges, for which reason it was excluded from this work. Periodic calculations were made for with both a 2 × 2 × 2 and a 3 × 3 × 3 *MgO* supercell.

The local properties of the electronic structure of *MgO* in periodic and embedded cluster models are presented in Table 2 Comparison of local properties between periodic calculations with different supercell sizes shows discrepancy for the individual descriptors, which one can consider as a threshold. For all local properties the difference is either in the second or in the third decimal point.

**TABLE 2:** Electronic structure of clusters with Mg- on O- center. Table showing the convergences of Mulliken charges (*Q*), Wiberg bond indices (*W*), covalency (*C*), total valency (*V*) and integrated density difference Δ*ρ* (multiplied by a factor of 10^3^) with respect to periodic values. * indicates that the value is undefined for the particular cluster.

	Cluster							Periodic	
Property	Mg-I	Mg-II	Mg-III	Mg-IV	O-I	O-II	O-III	2 × 2 × 2	3 × 3 × 3
$Q_{Mg^{\left(i\right)}}$	1.024	1.127	1.276	1.166	1.867	1.097	1.120	±1.153	±1.159
$Q_{O^{\left(i\right)}}$	−1.837	−1.183	−1.189	−1.167	−1.200	−1.204	−1.175		
$W_{Mg^{\left(i\right)}O^{\left(i\right)}}$	0.283	0.244	0.209	0.255	0.283	0.221	0.229	0.256	0.265
$W_{O^{\left(i\right)}Mg^{\left(o\right)}}$	*	0.236	0.227	0.241	*	0.265	0.259		
$C_{Mg^{\left(i\right)}}$	1.696	1.536	1.285	1.459	0.256	1.588	1.540	1.497	1.454
$C_{O^{\left(i\right)}}$	0.302	1.443	1.420	1.425	1.429	1.408	1.408	1.460	1.446
$V_{Mg^{\left(i\right)}}$	2.178	2.132	2.071	2.105	1.999	2.148	2.129	2.123	2.095
$V_{O^{\left(i\right)}}$	1.995	2.107	2.095	2.080	2.111	2.099	2.074	2.094	2.089
10^3^*ρ ^*RMSD*^	13.02	12.38	3.130	2.510	15.53	6.890	5.181	5.986	Ref

The smallest clusters, **Mg-I** and **O-I** have only one (central) atom with a proper atomic surrounding, thus, it is rather expected that the electronic structure even for the central region of these clusters is different within **Mg-I**–**Mg-IV** and **O-I**–**O-III** series. The atomic charge on the central *Mg* atom in **Mg-I** is 1.02e, while for larger clusters it is in the range 1.13–1.28 e. For the smallest clusters (**Mg-I** and **O-I**) the charges on the central atoms are ‘non symmetric’ for *Mg* in **Mg-I** it is 1.02 e, but for *O* in **O-I** it is −1.20 e. The difference between atomic charges on the central atoms for the larger clusters in by order of magnitude smaller (1.166 e for **Mg-IV** *vs.* −1.175 e for **O-III**). If we exclude the smallest clusters from consideration, the atomic charges are rather close to the charges in the periodic model. The difference between the absolute values of atomic charges on the central atom and on the nearest neighbor is also reducing with the increase of the cluster size. Increasing the cluster sizes from **Mg-I** to **Mg-IV** and from **O-I** to **O-III** does not result in a monotonic change in atomic charges. Instead of correlating with whether the central atom is *Mg* or *O*, the atomic charges correlate with the formal charge of the cluster. Thus, for a monotonic charge the clusters should be viewed in the order of **Mg-I** to **O-II** and finally **Mg-III**, which are all anionic clusters.

On the other hand, the variations of the atomic charges are relatively small: 0.02 e for oxygen atom in clusters **O-II** and **O-III**, and 0.1 e for *Mg* in clusters **Mg-II**–**Mg-IV**. The largest deviation is observed for **Mg-III** cluster, which in the quantum part has the largest negative charge.

The bond orders, also known as Wiberg indices, between *Mg* and *O* show only a small variation between clusters and they are similar to periodic values. Again the largest difference from the general trend is observed for the **Mg-III** cluster. Considering that the lowest bond index for *Mg*–*O* bond and largest atomic charge on *Mg* atom was obtained for the **Mg-III** cluster, we can conclude that this cluster is the has most ionic character of the clusters studied here. The covalency of central *Mg* atom in **Mg-IV** cluster (1.46) is almost the same as the covalency of *Mg* in periodic calculations (1.45). The covalency of *O* in **O-II** and **O-III** (1.41) is also close to corresponding value (1.45) in periodic calculations. Total valence of *Mg* and *O* in all clusters, except the smallest ones, is close to the corresponding values in periodic calculations.

**FIGURE 3:**
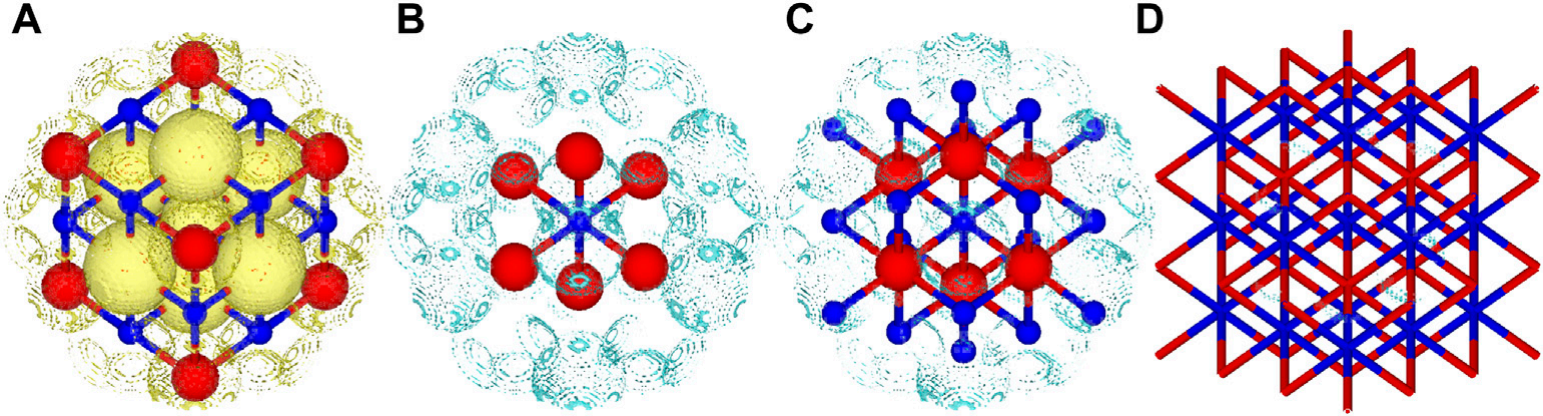
Total and difference density plots for *MgO*. **(A)** Total density retained from the 3 × 3 × 3 periodic calculation of *MgO*, only a small part of the total structure visualised for brevity, **(B)** density difference between the ${\left[MgO_{6}\right]}^{10-}$ -cluster and the 3 × 3 × 3 calculation, **(C)** density difference between the ${\left[MgO_{6}Mg_{18}\right]}^{26+}$ -cluster and the 3 × 3 × 3 calculation and **(D)** density difference between the ${\left[MgO_{6}Mg_{18}O_{33}\right]}^{50-}$ -cluster and the 3 × 3 × 3 calculation. A wireframe model was used for **(D)** in order to make the isosurface more visible. Isosurface values of 0.04 e/ $a_{0}^{3}$ . Yellow isosurfaces correspond to positive values and cyan to negative values. Blue spheres correspond to *Mg* and red spheres to *O*.

In contrast, the RMSD of the electron density difference gave a smooth convergence towards the periodic limit for the central *XY*_6_ unit. For **Mg-I**, **Mg-II** and **O-I** clusters, the error is in the range of 0.012–0.015 $e/a_{0}^{3}$ , but for larger clusters it is in the range of 0.002–0.005 $e/a_{0}^{3}$ , which is not larger than the corresponding difference between densities obtained for different supercell extensions. In Figure 3, the spatial electron density used in the comparison is visualised, along with density differences plots for the **Mg-I**, **Mg-II** and **Mg-III** clusters.

### 3.2 Local Electronic Structure of Ni:MgO

Studies of single-ion dopants has been a major undertaking for AIMP studies. In this section, the convergence of the local electronic structure of *Ni* and *O* in *Ni*: *MgO* is presented. As in the previous sections, the results will be written for all three clusters on the order of **Ni-I**, **Ni-II** and **Ni-III**. We should note here that the calculations of these clusters were made with UHF Hamiltonian with triplet spin multiplicity. Periodic calculations were made by substituting a single *Mg* with *Ni* in both the 2 × 2 × 2 and 3 × 3 × 3 *MgO* supercells.

The local properties of the electronic structure of *Ni*: *MgO* in periodic and embedding cluster model are presented in Table 3. The difference between 2 × 2 × 2 and 3 × 3 × 3 periodic calculations is slightly larger, compared to pure *MgO* due to interaction between *Ni* atoms. Cluster models with a single nickel atom is free from this problem. Similar to *MgO* clusters, the smallest cluster (**Ni-I**) differs the most from the other clusters. The atomic charge on *Ni* increases when increasing the cluster size to **Ni-II** and **Ni-III**, but drops for the **Ni-IV** cluster, and becomes similar to the atomic charge in both periodic models. The atomic charge on *Ni* is smaller than the corresponding charge on *Mg*. But at the same time the covalent bonds between *Ni*–*O* are stronger than *Mg*–*O* bond. That results in larger, in comparison to *Mg*, covalency of nickel atom and the total valency 2.3. The ‘elevated’ total valency for nickel atom is not an unexpected result, since Ni atom can be found in different valence states Evarestov et al. (1994).

**TABLE 3:** Electronic structure of clusters Ni:MgO. Table showing the convergences of Mulliken charges (*Q*), Wiberg bond indices (*W*), covalency (*C*), total valency (*V*) and integrated density difference Δ*ρ* (multiplied by a factor of 10^3^) with respect to periodic values. * indicates that the value is undefined for the particular cluster.

Property	Cluster				Periodic	
	Ni-I	Ni-II	Ni-III	Ni-IV	2 × 2 × 2	3 × 3 × 3
*Q*_*Ni*_	0.619	0.688	0.865	0.791	0.822	0.779
$Q_{O^{\left(i\right)}}$	−1.770	−1.115	−1.126	−1.106	−1.142	−1.155
$W_{NiO^{\left(i\right)}}$	0.386	0.348	0.305	0.335	0.325	0.340
$W_{O^{\left(i\right)}Mg^{\left(o\right)}}$	*	0.228	0.219	0.271	0.258	0.265
*C*_*Ni*_	2.315	2.305	1.996	2.096	2.046	2.105
$C_{O^{\left(i\right)}}$	0.412	1.534	1.504	1.504	1.472	1.454
*V*_*Ni*_	2.470	2.495	2.319	2.361	2.336	2.361
$V_{O^{\left(i\right)}}$	1.988	2.120	2.106	2.090	2.095	2.089
10^3^*ρ ^*RMSD*^	12.87	12.43	3.365	2.794	5.986	Ref

Comparison of electron density shows a similar trend as for *MgO* clusters. The electron density in **Ni-I** and **Ni-II** clusters is slightly different from density in the periodic calculations. But for the **Ni-III** and **Ni-IV** clusters, the difference is negligibly small.

**FIGURE 4:**
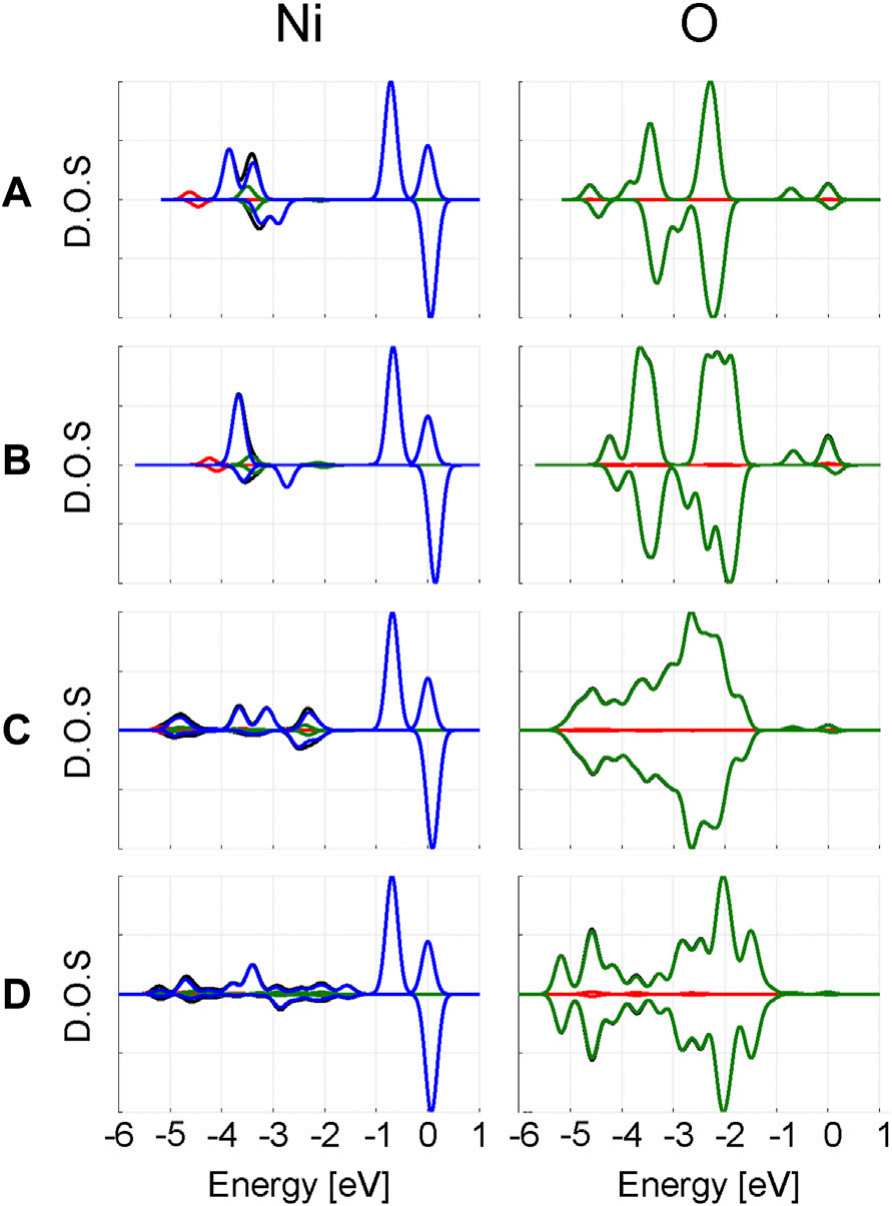
Projected density of states from clusters **(A)** ${\left[{MgO}_{6}\right]}^{10-}$ , **(B)** ${\left[{MgO}_{6}{Mg}_{18}\right]}^{26+}$ , **(C)** ${\left[{MgO}_{6}{Mg}_{18}O_{38}\right]}^{50-}$ and **(D)** periodic PBE calculations on *Ni*: *MgO*. The *α* spin channels are plotted with positive amplitudes and *β* spin channels with negative amplitudes. The Fermi level of the *α* spin channel is set as zero. D.O.S stands for density of states and is given in arbitrary units. The colour scheme of the PDOS is as follows: black—total, red—s, green—p and blue—d. The PDOS were obtained *via* Gaussian smearing of the canonical molecular orbital energies, using a smearing width of 1 meV.

In Figure 4, the projected density of states (PDOS) for *Ni* and *O* are plotted. The d-states of *Ni* are independent of the computational model used. In the *α* spin channel, the double peak closest to the Fermi level corresponds to the split between the *t*_2*g*_ and *e*_*g*_ *Ni* d-orbitals. Since no *e*_*g*_ orbital is occupied in the *β* channel, there is only a single *t*_2*g*_ peak near the Fermi level. The difference between the *t*_2*g*_ and *e*_*g*_ *α* peaks change as 0.72 eV, 0.67–0.69 eV when increasing the model size. From the periodic calculations, a split of 0.70 eV is predicted.

In addition to these two major peaks, there are several minor peaks at lower energies originating from the *Ni* d-orbitals. Comparison with the *O* PDOS at the same energy region suggests these are primarily caused by the *Ni* d-*functions* acting as additional polarizing basis functions for the oxygen p-band. Overall, larger changes are visible in the *O* PDOS compared to the *Ni* PDOS; primarily a broadening of the PDOS is observed when increasing the cluster size. This is an expected consequence of including more “real” oxygens in the model.

The advantage of embedded cluster model in comparison to periodic model is that all methods used in molecular quantum chemistry can be applied. It is very important for the properties, involving excited states, in particular excitation spectra. In Larsson et al. (2022) we presented the vibronic d–d transition in *Ni*: *MgO*, which was modeled as **Ni-II** embedded cluster. There, it was demonstrated that when using multiconfigurational theory and appropriate basis sets (ANO-RCC), the experimental spectrum of *Ni*: *MgO* could be reproduced to within 1000 cm^−1^ on average.

Obviously, one advantage of a material such as *MgO* is that, in comparison to transition metal oxides (e.g., *TiO*_2_), it is a relatively simple material. Adding additional layers of *Mg* or *O* does not increase the number of orbitals that might influence, for instance, the spectrum of single ion dopants. In materials like *TiO*_2_, the presence of valence d-orbitals, even if they are formally empty, potentially complicates the picture dramatically. More extensive studies are necessary to verify the applicability of multiconfigurational methods in such systems. At the present, the AIMP methodology is presented as a straightforward and accurate way of modelling ionic materials made from s or p group elements.

### 3.3 Computational Costs

The computational cost to compute DFT density for these clusters consists of computing the integrals (in the compact form of RI/CD technique implemented in OpenMolcas) and self consistent field iterations. Even with the use of RI/CD technique the size of the temporary disk space is large and can easily reach several hundreds of Gb. The calculation of integrals is approximately one third of the total time. The size of AIMPs and electrostatic field has no effect on the timing. All calculations were performed at Tetralith cluster, with Intel Xeon Gold 6130 CPU and SSD hard drive. The calculations with 8 cores of **Mg-I** cluster take less than a minute, **Mg-II**—2 min, **Mg-III**—14 min and finally **Mg-IV**—2 h. The total timing also depends on the convergence of the optimal wavefunction. We should note here that a calculation with the periodic boundary conditions (performed with CP2K code with the same basis set and the same computational setup) takes even longer time. The computational resources will increase if a more accurate basis will be used, see Table 1. Using multiconfigurational theory for accurate calculations of the electronic structure will increase the timing in the order of magnitude.

## 4 Conclusion

Using PBE Hamiltonian and a relatively small basis set we investigated the convergence of the electron density with an increase of the size of embedded clusters. The results shown herein demonstrate that good agreement in both local properties and spatial electron density can be achieved for moderately sized clusters using AIMP embedding. Using the spatial electron density to assess the clusters gives a clearer convergence with increasing cluster size, compared to the local properties. One advantage with the local properties, however, is that they are generally faster to compute and therefore give an important first quality check of the clusters.

The smallest clusters considered accurate here (**Mg-II**, **O-II** and **Ni-II**) all achieve the following two criteria: 1) no single element in the material is described only by AIMPs and 2) in the central region, the co-ordination of all ions are completely saturated. This suggests that in future studies, minimal clusters should be constructed based on such criteria.

While the largest discrepancies were noted for the most anionic clusters (**Mg-III** and **Ni-III**, both with a formal charge of −50 e), the values are seemingly not far enough from periodic values to completely invalidate the use of anionic clusters with AIMPs. Even more anionic clusters, however, seem to give poor convergence and are thus discouraged from use in production calculations.

## Data Availability

The original contributions presented in the study are included in the article/Supplementary Material, further inquiries can be directed to the corresponding author.
