# Supplementary material for: Convergence of Electronic Structure Properties in Ionic Oxides Within a Fragment Approach
Source: Front Chem. 2022 Jul 15;10:951144. doi: 10.3389/fchem.2022.951144 (PMC9334522; doi:10.3389/fchem.2022.951144)
Supplement: Supplementary file 1 [file DataSheet1.PDF]

Supplementary Material
Convergence of electronic structure properties in ionic oxides within a fragment approach

1 SUPPLEMENTARY DATA

1.1 AIMP library used in the study

```
/Mg.ECP.Larsson.10s7p.1s1p.0e-PBE0-MgO(bulk)
Citation line
Embedding AIMP parametrised with SCEPIC. Valence functions added.
   2.0     1
* l=0-type functions
    10     1
   5220.199534   771.32169872   173.23388715   48.045521313
  24.982026475    14.60201368   2.5092153563  0.88252110745
 0.087759105802  0.033937958572
-0.001665634347
-0.010954227130
-0.053735209823
-0.139829936819
-0.072553335085
-0.130272969618
 0.562197998803
 0.541112807453
 0.036742850720
-0.019269674455
* l=1-type functions
     7     1
  100.96369265   23.089228108   6.9566580373   2.2630606101
 0.72252924357  0.18914796195  0.053768755187
 0.014640719915
 0.095666861823
 0.307647073581
 0.498722379598
 0.308322412860
 0.022942974202
 0.006796837187
M1
  9
 139740.811798   9905.4785109  1503.92320821  338.733905111
 97.7049504037  30.1569143173  10.8059336287  3.96675156925
 0.864304065931
```

```
 0.059328244872  0.121223823647  0.248472315053  0.456526773793
 0.614945479858  0.543148740863  0.916405079131  1.779179372574
 0.260770170210
M2
  0
COREREP
  1.0
PROJOP
  1
  10 2 2 2
   93.746  6.066
   5220.199534   771.32169872   173.23388715   48.045521313
  24.982026475    14.60201368   2.5092153563  0.88252110745
 0.087759105802  0.033937958572
 0.006493763224 -0.001665634347
 0.043002913206 -0.010954227130
 0.190091537578 -0.053735209823
 0.465375061769 -0.139829936819
 0.027706024143 -0.072553335085
 0.399617960947 -0.130272969618
 0.031607529793  0.562197998803
-0.009945375051  0.541112807453
 0.002396105007  0.036742850720
-0.001274243799 -0.019269674455
  7 1 6
    3.486
  100.96369265   23.089228108   6.9566580373   2.2630606101
 0.72252924357  0.18914796195  0.053768755187
 0.014640719915
 0.095666861823
 0.307647073581
 0.498722379598
 0.308322412860
 0.022942974202
 0.006796837187
Spectral Representation Operator
Core primitive basis
Exchange
End of Spectral Representation Operator

/Mg.ECP.Larsson.0s.0s.0e-PBE0-MgO(bulk)
Citation line
Embedding AIMP parametrised with SCEPIC.
   2.0     0
* l=0-type functions
```

```
     0     0
M1
  9
 139740.811798   9905.4785109  1503.92320821  338.733905111
 97.7049504037  30.1569143173  10.8059336287  3.96675156925
 0.864304065931
 0.059328244872  0.121223823647  0.248472315053  0.456526773793
 0.614945479858  0.543148740863  0.916405079131  1.779179372574
 0.260770170210
M2
  0
COREREP
  1.0
PROJOP
  1
  10 2 2 2
   93.746  6.066
   5220.199534   771.32169872   173.23388715   48.045521313
  24.982026475    14.60201368   2.5092153563  0.88252110745
 0.087759105802  0.033937958572
 0.006493763224 -0.001665634347
 0.043002913206 -0.010954227130
 0.190091537578 -0.053735209823
 0.465375061769 -0.139829936819
 0.027706024143 -0.072553335085
 0.399617960947 -0.130272969618
 0.031607529793  0.562197998803
-0.009945375051  0.541112807453
 0.002396105007  0.036742850720
-0.001274243799 -0.019269674455
  7 1 6
    3.486
  100.96369265   23.089228108   6.9566580373   2.2630606101
 0.72252924357  0.18914796195  0.053768755187
 0.014640719915
 0.095666861823
 0.307647073581
 0.498722379598
 0.308322412860
 0.022942974202
 0.006796837187
Spectral Representation Operator
Core primitive basis
Exchange
End of Spectral Representation Operator
```

```
/O.ECP.Larsson.7s4p.1s1p.0e-PBE0-MgO(bulk)
Citation line
Embedding AIMP parametrised with SCEPIC. Valence functions added.
  -2.0     1
* l=0-type functions
     7     1
  2256.4894524   338.49913922   76.906383305   21.408862113
  6.6523625585  0.81216936025  0.25588704958
-0.001263312142
-0.009317536841
-0.040175570280
-0.135875506759
-0.150469540085
 0.561818759337
 0.540775131282
* l=1-type functions
     4     1
  17.753536645   3.8671566736   1.0487786113  0.27651001706
 0.037771426036
 0.200106928283
 0.383633384714
 0.616292237152
M1
  8
 46699.3344079  1821.75346453   232.41725884  50.1950677746
 13.5747696349  4.44387991646  1.60198675438  0.547233084104
-0.072492184897 -0.197248546592 -0.420030545100 -0.640176686713
-0.479145527194 -0.703280200719 -1.356840021962 -1.130786286822
M2
  0
COREREP
  1.0
PROJOP
  1
  7 2 2 2
   37.906  1.467
  2256.4894524   338.49913922   76.906383305   21.408862113
  6.6523625585  0.81216936025  0.25588704958
 0.006050855764 -0.001263312142
 0.041880142609 -0.009317536841
 0.184919644856 -0.040175570280
 0.457135474451 -0.135875506759
 0.441843726966 -0.150469540085
 0.035819401348  0.561818759337
```

```
-0.012491996568  0.540775131282
   4 1 6
     0.331
   17.753536645   3.8671566736   1.0487786113  0.27651001706
  0.037771426036
  0.200106928283
  0.383633384714
  0.616292237152
 Spectral Representation Operator
 Core primitive basis
 Exchange
 End of Spectral Representation Operator

 /O.ECP.Larsson.0s.0s.0e-PBE0-MgO(bulk)
 Citation line
 Embedding AIMP parametrised with SCEPIC.
   -2.0     0
 * l=0-type functions
      0     0
 M1
   8
  46699.3344079  1821.75346453   232.41725884  50.1950677746
  13.5747696349  4.44387991646  1.60198675438  0.547233084104
-0.072492184897 -0.197248546592 -0.420030545100 -0.640176686713
-0.479145527194 -0.703280200719 -1.356840021962 -1.130786286822
 M2
   0
 COREREP
   1.0
 PROJOP
   1
   7 2 2 2
    37.906  1.467
   2256.4894524   338.49913922   76.906383305   21.408862113
   6.6523625585  0.81216936025  0.25588704958
  0.006050855764 -0.001263312142
  0.041880142609 -0.009317536841
  0.184919644856 -0.040175570280
  0.457135474451 -0.135875506759
  0.441843726966 -0.150469540085
  0.035819401348  0.561818759337
-0.012491996568  0.540775131282
   4 1 6
     0.331
   17.753536645   3.8671566736   1.0487786113  0.27651001706
```

```
 0.037771426036
 0.200106928283
 0.383633384714
 0.616292237152
Spectral Representation Operator
Core primitive basis
Exchange
End of Spectral Representation Operator


/Mg.ECP.Larsson.10s7p.1s1p.0e-PBE-MgO(bulk)
Citation line
Embedding AIMP parametrised with SCEPIC. Valence functions added.
   2.0     1
* l=0-type functions
    10     1
   5220.199534   771.32169872   173.23388715   48.045521313
  24.982026475    14.60201368   2.5092153563  0.88252110745
 0.087759105802  0.033937958572
-0.001668673956
-0.010970162157
-0.053850086073
-0.139666319853
-0.072502486915
-0.129270063857
 0.559164701601
 0.540246082966
 0.048148308509
-0.024127223719
* l=1-type functions
     7     1
  100.96369265   23.089228108   6.9566580373   2.2630606101
 0.72252924357  0.18914796195  0.053768755187
 0.014785957888
 0.096278620972
 0.308570869530
 0.495854753167
 0.306582496185
 0.032351222431
 0.007841039556
M1
  9
 139740.811798   9905.4785109  1503.92320821  338.733905111
 97.7049504037  30.1569143173  10.8059336287  3.96675156925
 0.873034410031
```

```
 0.059307354326  0.121182953447  0.248393835373  0.456305362105
 0.614445931282  0.544283786374  0.915983011030  1.769618430246
 0.270479335818
M2
  0
COREREP
  1.0
PROJOP
  1
  10 2 2 2
   92.344  5.647
   5220.199534   771.32169872   173.23388715   48.045521313
  24.982026475    14.60201368   2.5092153563  0.88252110745
 0.087759105802  0.033937958572
 0.006502848974 -0.001668673956
 0.043078435538 -0.010970162157
 0.190164129246 -0.053850086073
 0.465201850614 -0.139666319853
 0.025670895546 -0.072502486915
 0.402003451265 -0.129270063857
 0.031606307792  0.559164701601
-0.010278552985  0.540246082966
 0.002479087809  0.048148308509
-0.001314033907 -0.024127223719
  7 1 6
    3.201
  100.96369265   23.089228108   6.9566580373   2.2630606101
 0.72252924357  0.18914796195  0.053768755187
 0.014785957888
 0.096278620972
 0.308570869530
 0.495854753167
 0.306582496185
 0.032351222431
 0.007841039556
Spectral Representation Operator
Core primitive basis
Exchange
End of Spectral Representation Operator

/Mg.ECP.Larsson.0s.0s.0e-PBE-MgO(bulk)
Citation line
Embedding AIMP parametrised with SCEPIC.
   2.0     0
* l=0-type functions
```

```
     0     0
M1
  9
 139740.811798   9905.4785109  1503.92320821  338.733905111
 97.7049504037  30.1569143173  10.8059336287  3.96675156925
 0.873034410031
 0.059307354326  0.121182953447  0.248393835373  0.456305362105
 0.614445931282  0.544283786374  0.915983011030  1.769618430246
 0.270479335818
M2
  0
COREREP
  1.0
PROJOP
  1
  10 2 2 2
   92.344  5.647
   5220.199534   771.32169872   173.23388715   48.045521313
  24.982026475    14.60201368   2.5092153563  0.88252110745
 0.087759105802  0.033937958572
 0.006502848974 -0.001668673956
 0.043078435538 -0.010970162157
 0.190164129246 -0.053850086073
 0.465201850614 -0.139666319853
 0.025670895546 -0.072502486915
 0.402003451265 -0.129270063857
 0.031606307792  0.559164701601
-0.010278552985  0.540246082966
 0.002479087809  0.048148308509
-0.001314033907 -0.024127223719
  7 1 6
    3.201
  100.96369265   23.089228108   6.9566580373   2.2630606101
 0.72252924357  0.18914796195  0.053768755187
 0.014785957888
 0.096278620972
 0.308570869530
 0.495854753167
 0.306582496185
 0.032351222431
 0.007841039556
Spectral Representation Operator
Core primitive basis
Exchange
End of Spectral Representation Operator
```

```
/O.ECP.Larsson.7s4p.1s1p.0e-PBE-MgO(bulk)
Citation line
Embedding AIMP parametrised with SCEPIC. Valence functions added.
  -2.0     1
* l=0-type functions
     7     1
  2256.4894524   338.49913922   76.906383305   21.408862113
  6.6523625585  0.81216936025  0.25588704958
-0.001276221597
-0.009407268496
-0.040618844157
-0.136811427361
-0.149856498093
 0.563159356434
 0.539251617884
* l=1-type functions
     4     1
  17.753536645   3.8671566736   1.0487786113  0.27651001706
 0.038406999773
 0.203052654402
 0.377784830036
 0.620078547619
M1
  8
 46699.3344079  1821.75346453   232.41725884  50.1950677746
 13.5747696349  4.44387991646  1.60198675438  0.547233084104
-0.072595452409 -0.196769179947 -0.420534241626 -0.639764189024
-0.480402155023 -0.707126086905 -1.341915296964 -1.140893398101
M2
  0
COREREP
  1.0
PROJOP
  1
  7 2 2 2
   36.988  1.224
  2256.4894524   338.49913922   76.906383305   21.408862113
  6.6523625585  0.81216936025  0.25588704958
 0.006063926350 -0.001276221597
 0.041956312427 -0.009407268496
 0.185243883306 -0.040618844157
 0.455349595315 -0.136811427361
 0.443189386323 -0.149856498093
 0.036859588806  0.563159356434
```

```
-0.013279574698  0.539251617884
  4 1 6
    0.191
  17.753536645   3.8671566736   1.0487786113  0.27651001706
 0.038406999773
 0.203052654402
 0.377784830036
 0.620078547619
Spectral Representation Operator
Core primitive basis
Exchange
End of Spectral Representation Operator

/O.ECP.Larsson.0s.0s.0e-PBE-MgO(bulk)
Citation line
Embedding AIMP parametrised with SCEPIC.
  -2.0     0
* l=0-type functions
     0     0
M1
  8
 46699.3344079  1821.75346453   232.41725884  50.1950677746
 13.5747696349  4.44387991646  1.60198675438  0.547233084104
-0.072595452409 -0.196769179947 -0.420534241626 -0.639764189024
-0.480402155023 -0.707126086905 -1.341915296964 -1.140893398101
M2
  0
COREREP
  1.0
PROJOP
  1
  7 2 2 2
   36.988  1.224
  2256.4894524   338.49913922   76.906383305   21.408862113
  6.6523625585  0.81216936025  0.25588704958
 0.006063926350 -0.001276221597
 0.041956312427 -0.009407268496
 0.185243883306 -0.040618844157
 0.455349595315 -0.136811427361
 0.443189386323 -0.149856498093
 0.036859588806  0.563159356434
-0.013279574698  0.539251617884
  4 1 6
    0.191
  17.753536645   3.8671566736   1.0487786113  0.27651001706
```

```
 0.038406999773
 0.203052654402
 0.377784830036
 0.620078547619
Spectral Representation Operator
Core primitive basis
Exchange
End of Spectral Representation Operator


/Mg.ECP.Larsson.10s7p.1s1p.0e-HF-MgO(bulk)
Citation line
Embedding AIMP parametrised with SCEPIC. Valence functions added.
   2.0     1
* l=0-type functions
    10     1
   5220.199534   771.32169872   173.23388715   48.045521313
  24.982026475    14.60201368   2.5092153563  0.88252110745
 0.087759105802  0.033937958572
-0.001656373594
-0.010903010138
-0.053388167827
-0.139922566610
-0.074067304397
-0.131233035245
 0.570092745168
 0.540458505043
 0.015300844796
-0.010132987569
* l=1-type functions
     7     1
  100.96369265   23.089228108   6.9566580373   2.2630606101
 0.72252924357  0.18914796195  0.053768755187
 0.014190014173
 0.094119410979
 0.305534197468
 0.504444226111
 0.311667127047
 0.004998930866
 0.004337133298
M1
  9
 139740.811798   9905.4785109  1503.92320821  338.733905111
 97.7049504037  30.1569143173  10.6978742924  3.96675156925
 0.916686130533
```

```
 0.059356840400  0.121300842398  0.248534444133  0.457119432870
 0.615460156550  0.542538021833  0.928671406245  1.769007915028
 0.258010940544
M2
  0
COREREP
  1.0
PROJOP
  1
  10 2 2 2
   97.940  7.254
   5220.199534   771.32169872   173.23388715   48.045521313
  24.982026475    14.60201368   2.5092153563  0.88252110745
 0.087759105802  0.033937958572
 0.006470633150 -0.001656373594
 0.042798067730 -0.010903010138
 0.189962810608 -0.053388167827
 0.465472465565 -0.139922566610
 0.034347367180 -0.074067304397
 0.392281698062 -0.131233035245
 0.031575707167  0.570092745168
-0.008997902354  0.540458505043
 0.002172828565  0.015300844796
-0.001166839018 -0.010132987569
  7 1 6
    4.274
  100.96369265   23.089228108   6.9566580373   2.2630606101
 0.72252924357  0.18914796195  0.053768755187
 0.014190014173
 0.094119410979
 0.305534197468
 0.504444226111
 0.311667127047
 0.004998930866
 0.004337133298
Spectral Representation Operator
Core primitive basis
Exchange
End of Spectral Representation Operator

/Mg.ECP.Larsson.0s.0s.0e-HF-MgO(bulk)
Citation line
Embedding AIMP parametrised with SCEPIC.
   2.0     0
* l=0-type functions
```

```
     0     0
M1
  9
 139740.811798   9905.4785109  1503.92320821  338.733905111
 97.7049504037  30.1569143173  10.6978742924  3.96675156925
 0.916686130533
 0.059356840400  0.121300842398  0.248534444133  0.457119432870
 0.615460156550  0.542538021833  0.928671406245  1.769007915028
 0.258010940544
M2
  0
COREREP
  1.0
PROJOP
  1
  10 2 2 2
   97.940  7.254
   5220.199534   771.32169872   173.23388715   48.045521313
  24.982026475    14.60201368   2.5092153563  0.88252110745
 0.087759105802  0.033937958572
 0.006470633150 -0.001656373594
 0.042798067730 -0.010903010138
 0.189962810608 -0.053388167827
 0.465472465565 -0.139922566610
 0.034347367180 -0.074067304397
 0.392281698062 -0.131233035245
 0.031575707167  0.570092745168
-0.008997902354  0.540458505043
 0.002172828565  0.015300844796
-0.001166839018 -0.010132987569
  7 1 6
    4.274
  100.96369265   23.089228108   6.9566580373   2.2630606101
 0.72252924357  0.18914796195  0.053768755187
 0.014190014173
 0.094119410979
 0.305534197468
 0.504444226111
 0.311667127047
 0.004998930866
 0.004337133298
Spectral Representation Operator
Core primitive basis
Exchange
End of Spectral Representation Operator
```

```
/O.ECP.Larsson.7s4p.1s1p.0e-HF-MgO(bulk)
Citation line
Embedding AIMP parametrised with SCEPIC. Valence functions added.
  -2.0     1
* l=0-type functions
     7     1
  2256.4894524   338.49913922   76.906383305   21.408862113
  6.6523625585  0.81216936025  0.25588704958
-0.001230260766
-0.009106725444
-0.038960216882
-0.133917090781
-0.151773572079
 0.559890479110
 0.543177702712
* l=1-type functions
     4     1
  17.753536645   3.8671566736   1.0487786113  0.27651001706
 0.035966212817
 0.193414758612
 0.396506456566
 0.608127226684
M1
  8
 46699.3344079  1821.75346453   232.41725884  50.1950677746
 13.5747696349  4.44387991646  1.60198675438  0.547233084104
-0.072481944299 -0.197090981633 -0.420223876978 -0.640805814358
-0.475784762546 -0.694797983878 -1.390072075491 -1.108742560817
M2
  0
COREREP
  1.0
PROJOP
  1
  7 2 2 2
   40.600  2.096
  2256.4894524   338.49913922   76.906383305   21.408862113
  6.6523625585  0.81216936025  0.25588704958
 0.006018950409 -0.001230260766
 0.041692075418 -0.009106725444
 0.184097128487 -0.038960216882
 0.461863085989 -0.133917090781
 0.438320874977 -0.151773572079
 0.032814015588  0.559890479110
```

```
-0.010307659273  0.543177702712
   4 1 6
     0.664
   17.753536645   3.8671566736   1.0487786113  0.27651001706
  0.035966212817
  0.193414758612
  0.396506456566
  0.608127226684
 Spectral Representation Operator
 Core primitive basis
 Exchange
 End of Spectral Representation Operator

 /O.ECP.Larsson.0s.0s.0e-HF-MgO(bulk)
 Citation line
 Embedding AIMP parametrised with SCEPIC.
   -2.0     0
 * l=0-type functions
      0     0
 M1
   8
  46699.3344079  1821.75346453   232.41725884  50.1950677746
  13.5747696349  4.44387991646  1.60198675438  0.547233084104
-0.072481944299 -0.197090981633 -0.420223876978 -0.640805814358
-0.475784762546 -0.694797983878 -1.390072075491 -1.108742560817
 M2
   0
 COREREP
   1.0
 PROJOP
   1
   7 2 2 2
    40.600  2.096
   2256.4894524   338.49913922   76.906383305   21.408862113
   6.6523625585  0.81216936025  0.25588704958
  0.006018950409 -0.001230260766
  0.041692075418 -0.009106725444
  0.184097128487 -0.038960216882
  0.461863085989 -0.133917090781
  0.438320874977 -0.151773572079
  0.032814015588  0.559890479110
-0.010307659273  0.543177702712
   4 1 6
     0.664
   17.753536645   3.8671566736   1.0487786113  0.27651001706
```

```
 0.035966212817
 0.193414758612
 0.396506456566
 0.608127226684
Spectral Representation Operator
Core primitive basis
Exchange
End of Spectral Representation Operator
```

1.2 example of an input file for cluster calculation

```
&GATEWAY
  Basis set
    Mg.PC-1
    Mg1  0.0000000000        0.0000000000        0.0000000000  Angstrom
  End of Basis
  Basis set
    O.PC-1
    O1   0.0000000000      2.1282420000  0.0000000000  Angstrom
    O2   0.0000000000      0.0000000000  2.1282420000  Angstrom
    O3   2.1282420000      0.0000000000  0.0000000000  Angstrom
    O4  -2.1282420000      0.0000000000  0.0000000000  Angstrom
    O5   0.0000000000     -2.1282420000  0.0000000000  Angstrom
    O6   0.0000000000      0.0000000000 -2.1282420000  Angstrom
  End of Basis
  Basis set
    Mg.ECP.Larsson.0s.0s.0e-PBE-MgO(bulk) / AIMPLIB
    pseudocharge
    A1   0.0000000000      2.1282420000  2.1282420000  Angstrom
    A2   2.1282420000      0.0000000000  2.1282420000  Angstrom
    A3   2.1282420000      2.1282420000  0.0000000000  Angstrom
    A4  -2.1282420000      0.0000000000  2.1282420000  Angstrom
    A5  -2.1282420000      2.1282420000  0.0000000000  Angstrom
    A6   0.0000000000     -2.1282420000  2.1282420000  Angstrom
    A7   2.1282420000     -2.1282420000  0.0000000000  Angstrom
    A8  -2.1282420000     -2.1282420000  0.0000000000  Angstrom
    A9   0.0000000000      2.1282420000 -2.1282420000  Angstrom
    A10  2.1282420000      0.0000000000 -2.1282420000  Angstrom
    A11 -2.1282420000      0.0000000000 -2.1282420000  Angstrom
    A12  0.0000000000     -2.1282420000 -2.1282420000  Angstrom
    A13  0.0000000000      0.0000000000  4.2564840000  Angstrom
    A14  0.0000000000      4.2564840000  0.0000000000  Angstrom
    A15  4.2564840000      0.0000000000  0.0000000000  Angstrom
    A16 -4.2564840000      0.0000000000  0.0000000000  Angstrom
```

```
    A17  0.0000000000     -4.2564840000  0.0000000000  Angstrom
    A18  0.0000000000      0.0000000000 -4.2564840000  Angstrom
    A19  2.1282420000      2.1282420000  4.2564840000  Angstrom
    A20  2.1282420000      4.2564840000  2.1282420000  Angstrom
    A21  4.2564840000      2.1282420000  2.1282420000  Angstrom
    A22 -2.1282420000      2.1282420000  4.2564840000  Angstrom
    A23 -2.1282420000      4.2564840000  2.1282420000  Angstrom
    A24 -4.2564840000      2.1282420000  2.1282420000  Angstrom
    A25  2.1282420000     -2.1282420000  4.2564840000  Angstrom
    A26  2.1282420000     -4.2564840000  2.1282420000  Angstrom
    A27  4.2564840000     -2.1282420000  2.1282420000  Angstrom
    A28 -2.1282420000     -2.1282420000  4.2564840000  Angstrom
    A29 -2.1282420000     -4.2564840000  2.1282420000  Angstrom
    A30 -4.2564840000     -2.1282420000  2.1282420000  Angstrom
    A31  2.1282420000      2.1282420000 -4.2564840000  Angstrom
    A32  2.1282420000      4.2564840000 -2.1282420000  Angstrom
    A33  4.2564840000      2.1282420000 -2.1282420000  Angstrom
    A34 -2.1282420000      2.1282420000 -4.2564840000  Angstrom
    A35 -2.1282420000      4.2564840000 -2.1282420000  Angstrom
    A36 -4.2564840000      2.1282420000 -2.1282420000  Angstrom
    A37  2.1282420000     -2.1282420000 -4.2564840000  Angstrom
    A38  2.1282420000     -4.2564840000 -2.1282420000  Angstrom
    A39  4.2564840000     -2.1282420000 -2.1282420000  Angstrom
    A40 -2.1282420000     -2.1282420000 -4.2564840000  Angstrom
    A41 -2.1282420000     -4.2564840000 -2.1282420000  Angstrom
    A42 -4.2564840000     -2.1282420000 -2.1282420000  Angstrom
    A43  0.0000000000      4.2564840000  4.2564840000  Angstrom
    A44  4.2564840000      0.0000000000  4.2564840000  Angstrom
    A45  4.2564840000      4.2564840000  0.0000000000  Angstrom
    A46 -4.2564840000      0.0000000000  4.2564840000  Angstrom
    A47 -4.2564840000      4.2564840000  0.0000000000  Angstrom
    A48  0.0000000000     -4.2564840000  4.2564840000  Angstrom
    A49  4.2564840000     -4.2564840000  0.0000000000  Angstrom
    A50 -4.2564840000     -4.2564840000  0.0000000000  Angstrom
    A51  0.0000000000      4.2564840000 -4.2564840000  Angstrom
    A52  4.2564840000      0.0000000000 -4.2564840000  Angstrom
    A53 -4.2564840000      0.0000000000 -4.2564840000  Angstrom
    A54  0.0000000000     -4.2564840000 -4.2564840000  Angstrom
  End of Basis
  Basis set
    O.ECP.Larsson.0s.0s.0e-PBE-MgO(bulk) / AIMPLIB
    pseudocharge
    A55   2.1282420000        2.1282420000   2.1282420000  Angstrom
    A56  -2.1282420000        2.1282420000   2.1282420000  Angstrom
    A57   2.1282420000       -2.1282420000   2.1282420000  Angstrom
```

A58	-2.1282420000	-2.1282420000	2.1282420000	Angstrom
A59	2.1282420000	2.1282420000	-2.1282420000	Angstrom
A60	-2.1282420000	2.1282420000	-2.1282420000	Angstrom
A61	2.1282420000	-2.1282420000	-2.1282420000	Angstrom
A62	-2.1282420000	-2.1282420000	-2.1282420000	Angstrom
A63	2.1282420000	0.0000000000	4.2564840000	Angstrom
A64	0.0000000000	2.1282420000	4.2564840000	Angstrom
A65	2.1282420000	4.2564840000	0.0000000000	Angstrom
A66	0.0000000000	4.2564840000	2.1282420000	Angstrom
A67	4.2564840000	0.0000000000	2.1282420000	Angstrom
A68	4.2564840000	2.1282420000	0.0000000000	Angstrom
A69	-2.1282420000	0.0000000000	4.2564840000	Angstrom
A70	-2.1282420000	4.2564840000	0.0000000000	Angstrom
A71	-4.2564840000	0.0000000000	2.1282420000	Angstrom
A72	-4.2564840000	2.1282420000	0.0000000000	Angstrom
A73	0.0000000000	-2.1282420000	4.2564840000	Angstrom
A74	2.1282420000	-4.2564840000	0.0000000000	Angstrom
A75	0.0000000000	-4.2564840000	2.1282420000	Angstrom
A76	4.2564840000	-2.1282420000	0.0000000000	Angstrom
A77	-2.1282420000	-4.2564840000	0.0000000000	Angstrom
A78	-4.2564840000	-2.1282420000	0.0000000000	Angstrom
A79	2.1282420000	0.0000000000	-4.2564840000	Angstrom
A80	0.0000000000	2.1282420000	-4.2564840000	Angstrom
A81	0.0000000000	4.2564840000	-2.1282420000	Angstrom
A82	4.2564840000	0.0000000000	-2.1282420000	Angstrom
A83	-2.1282420000	0.0000000000	-4.2564840000	Angstrom
A84	-4.2564840000	0.0000000000	-2.1282420000	Angstrom
A85	0.0000000000	-2.1282420000	-4.2564840000	Angstrom
A86	0.0000000000	-4.2564840000	-2.1282420000	Angstrom
A87	2.1282420000	4.2564840000	4.2564840000	Angstrom
A88	4.2564840000	2.1282420000	4.2564840000	Angstrom
A89	4.2564840000	4.2564840000	2.1282420000	Angstrom
A90	-2.1282420000	4.2564840000	4.2564840000	Angstrom
A91	-4.2564840000	2.1282420000	4.2564840000	Angstrom
A92	-4.2564840000	4.2564840000	2.1282420000	Angstrom
A93	2.1282420000	-4.2564840000	4.2564840000	Angstrom
A94	4.2564840000	-2.1282420000	4.2564840000	Angstrom
A95	4.2564840000	-4.2564840000	2.1282420000	Angstrom
A96	-2.1282420000	-4.2564840000	4.2564840000	Angstrom
A97	-4.2564840000	-2.1282420000	4.2564840000	Angstrom
A98	-4.2564840000	-4.2564840000	2.1282420000	Angstrom
A99	2.1282420000	4.2564840000	-4.2564840000	Angstrom
A100	4.2564840000	2.1282420000	-4.2564840000	Angstrom
A101	4.2564840000	4.2564840000	-2.1282420000	Angstrom
A102	-2.1282420000	4.2564840000	-4.2564840000	Angstrom

```
    A103 -4.2564840000        2.1282420000  -4.2564840000  Angstrom
    A104 -4.2564840000        4.2564840000  -2.1282420000  Angstrom
    A105  2.1282420000       -4.2564840000  -4.2564840000  Angstrom
    A106  4.2564840000       -2.1282420000  -4.2564840000  Angstrom
    A107  4.2564840000       -4.2564840000  -2.1282420000  Angstrom
    A108 -2.1282420000       -4.2564840000  -4.2564840000  Angstrom
    A109 -4.2564840000       -2.1282420000  -4.2564840000  Angstrom
    A110 -4.2564840000       -4.2564840000  -2.1282420000  Angstrom
  End of Basis
  xfield = $CurrDir/$Project.xfield

&SEWARD

&SCF
  ksdft    = pbe
  occupied = 35

&GRID_IT
  total
  dense
```

Inputs for larger clusters are available from the authors by request

1.3 Basis set selection

Clusters, created by layers of ions, can have very high electronic charge in the quantum part, although the AIMPs and point charges compensate this charge. The selection of basis set for the quantum part is therefore important. For highly anionic species, there is a general consensus that diffuse basis functions are required to achieve accurate results. This was ignored in this study, in favour of using a more compact basis set to more easily reach larger cluster size. To quantify if this neglect should have any significant impact on the results, diffuse functions were added to the oxygens (using the aug-pc-1 basis set) in one of the larger anionic clusters (Mg-III). The influence of these basis functions were tested by computing the RMSD of the density, using the Mg-III/pc-1 density as reference. Only a very small deviation of $0.33 * 10^{-3}$ was observed, suggesting that diffuse functions will have no major impact on the local properties discussed in the present study. In accurate calculations of properties, however, the authors' recommend using basis sets containing diffuse functions.
